# Healthcare professionals and managers' participation in developing an intervention: A pre-intervention study in the elderly care context

**DOI:** 10.1186/1748-5908-4-21

**Published:** 2009-04-21

**Authors:** Isabelle Vedel, Matthieu De Stampa, Howard Bergman, Joel Ankri, Bernard Cassou, François Blanchard, Liette Lapointe

**Affiliations:** 1 Université de Versailles St-Quentin, Laboratoire Santé Vieillissement, AP-HP, Hôpital Sainte Perine, 49 rue Mirabeau 75016 Paris, France; 2 Solidage, McGill University – Université de Montréal Research Group on Frailty and Aging, 3755 Ch. Côte Ste Catherine, Montréal H3T 1B3, Québec, Canada; 3 Desautels Faculty of Management, McGill University, 1001 Sherbrooke St West Montreal, QC H3A 1G5, Canada; 4 Division of Geriatric Medicine, Jewish General Hospital, McGill University, 3755 Ch. Côte Ste Catherine, Montréal H3T 1B3, Québec, Canada; 5 Université de Reims Champagne Ardennes, Laboratoire Santé Publique, Vieillissement et troubles cognitifs et du comportement, Hôpital Maison Blanche 45, rue Cognacq-Jay 51092 Reims, France

## Abstract

**Background:**

In order to increase the chances of success in new interventions in healthcare, it is generally recommended to tailor the intervention to the target setting and the target professionals. Nonetheless, pre-intervention studies are rarely conducted or are very limited in scope. Moreover, little is known about how to integrate the results of a pre-intervention study into an intervention. As part of a project to develop an intervention aimed at improving care for the elderly in France, a pre-intervention study was conducted to systematically gather data on the current practices, issues, and expectations of healthcare professionals and managers in order to determine the defining features of a successful intervention.

**Methods:**

A qualitative study was carried out from 2004 to 2006 using a grounded theory approach and involving a purposeful sample of 56 healthcare professionals and managers in Paris, France. Four sources of evidence were used: interviews, focus groups, observation, and documentation.

**Results:**

The stepwise approach comprised three phases, and each provided specific results. In the first step of the pre-intervention study, we gathered data on practices, perceived issues, and expectations of healthcare professionals and managers. The second step involved holding focus groups in order to define the characteristics of a tailor-made intervention. The third step allowed validation of the findings. Using this approach, we were able to design and develop an intervention in elderly care that met the professionals' and managers' expectations.

**Conclusion:**

This article reports on an in-depth pre-intervention study that led to the design and development of an intervention in partnership with local healthcare professionals and managers. The stepwise approach represents an innovative strategy for developing tailored interventions, particularly in complex domains such as chronic care. It highlights the usefulness of seeking out the insight of healthcare professionalnd managers and emphasizes the need to intervene at different levels. Further research will be needed in order to develop a more thorough understanding of the impacts of such strategies on the final outcomes of intervention implementations.

## Background

Many different approaches have been tried to improve quality of care, but these efforts have often failed or, at best, they have had modest or partial impacts [1-10], with considerable variations in the observed effects within and across interventions [1,3,11,12]. These disappointing results have led to a series of recommendations. One of these recommendations is to adopt a phased approach to the development and evaluation of complex interventions [13-15]. According to the authors, interventions should be fully defined and developed before being evaluated. Also, interventions should be tailored to the target setting and the target professionals [3,11,12]. No strategy is inherently superior in all situations and there is no magic bullet [1,5,10]. Therefore, it has been recommended that the context be investigated and potential users be involved in the intervention development process in order to tailor the intervention to local conditions and incorporate user perspectives [1,5,11,16-18]. While this strategy is generally recognized as a condition for successful implementations, and even if some uncertainties remain [12,18,19], pre-intervention diagnostic analyses of the context and the needs of potential users are rarely performed. Indeed, implementation research has little to say about the intervention design process [20]. First, most interventions are solution-driven rather than needs-driven [18] and are designed with only a limited description of the characteristics of the targeted behaviour, the professionals, and the context [2,20]. Only a few studies of implementations have included a pre-intervention phase in order to tailor the intervention to its context [21]. These studies are often limited to the identification of potential barriers to implementation at the individual level, leaving the context at the organizational level under-explored [12,20,22]. Second, little is known about how to integrate pre-intervention study results into the features of the intervention [12,20]. Even when a pre-implementation study is performed, most interventions do not incorporate its specific findings into the design of the intervention itself [12,20].

Several key research questions about the intervention development process remain, including how to develop strategies for gathering data from potential users as well as how to incorporate the data into the characteristics of the intervention itself [18,21]. In other words, considerable work is still required on how to develop a pre-intervention study that will investigate current practices, issues, and the expectations of healthcare professionals and managers with an eye to determining the defining features of the intervention.

While improving and reorganizing elderly care in modern health systems has become a priority in order to cope with the specific challenges of meeting the needs of older persons [23-25], the gap between conceptual models of care and existing provider practice remains wide [1,24,26]. Implementing change in chronic care is particularly challenging, and failures are numerous [1,5]. Indeed, projects implementing integrated-care programs [27-32] have taken centre stage as a way to improve quality and efficiency in elderly care. Despite strong evidence of their efficacy in optimizing resource utilization and health and satisfaction levels among older persons [30,33], it has been difficult to diffuse and sustain these programs, in large part because of difficulties encountered securing the participation of healthcare professionals and, in particular, primary care physicians (PCPs) [24,26,27,30,31,34,35]. This can be linked to the lack of an in-depth understanding of the context or of partnerships with local providers. Indeed, these integrated care programs were generally developed without pre-intervention studies. Thus, in chronic care, we are still trying to understand how to tailor implementation strategies to their context [5].

As part of a project to develop and implement an intervention aimed at improving elderly care in France, we conducted a pre-intervention study that would systematically gather data on current practices, issues, and expectations of healthcare professionals and managers in order to determine the defining features of the intervention. This paper proposes an innovative strategy for developing interventions that take into account the context of care. It highlights the usefulness of seeking out the insight of healthcare professionals and managers when developing an intervention in a particularly complex domain such as chronic care. Finally, it emphasizes the need to intervene at different levels, such as deploying evidence-based protocols at the individual level, implementing collaborative practices at the team level, and integrating services at the organizational level.

## Methods

### Research design

The method used in the pre-intervention study was based on the grounded theory building approach described by Pandit [36]. Unlike generating a framework *a priori *and then testing it [37], applying grounded theory involves developing and validating a framework in an iterative process based on three basic components: concepts, categories and, finally, propositions. In this case, the propositions are the defining characteristics of the intervention. The qualitative study translated into a three-step project that lasted two and one-half years (January 2004 to June 2006), with different objectives in each step. In the first step, participants were recruited for interviews in order to identify their current practices, perceived issues, and broad expectations regarding elderly care. The content of the interviews was then analyzed. The second step involved holding four focus groups with the same participants to refine the findings and to define the expected key features of the intervention. The content of the focus group discussions was then iteratively analyzed and presented to the focus group. Finally, in the third step, the results were presented to all participants for discussion and validated using a questionnaire with a five-point Likert scale.

### Setting and sampling

The research was conducted in the sixteenth borough of Paris, which has the greatest concentration of older people in Paris (11.4% of the population being 75 and older). Every hospital and community-based health and social service in this borough was invited to participate. In each setting, potential participants were selected using a purposeful sampling strategy followed by a snowball sampling strategy [38,39] in order to ensure good representation of healthcare professionals and managers (Table 1), whom we had identified as the main stakeholders in the project. Fifty-eight participants were selected, contacted, and asked to participate in individual face-to-face interviews and focus groups; only two PCPs declined the invitation. All participants gave informed verbal consent, and approval was obtained from the University Versailles Saint Quentin research committee. As indicated in Table 1, the participants represented a sample of healthcare professionals and managers from various settings and types of practice (in health and social services, hospitals, and community-based organizations).

**Table 1:** Description of the participants in the interviews and four focus groups

Setting	Profession	Total (N = 56)
Community-based services	Primary care physician	8
	Psychiatrist	2
	Nurse	5
	Physiotherapist	1
	Auxiliary nurse	2
	Social worker	6
	Home care worker	2
	Administrator	5
Hospitals	Geriatrician	3
	Emergency physician	2
	Nurse	4
	Physiotherapist	3
	Social worker	5
	Administrator	4
Organizations funding services	Administrator	4

### Data collection

In order to enhance the internal validity of the data, four sources of evidence were used: interviews, focus groups, observation, and documentation.

### Interviews

Three researchers (IV, MDS, CM) conducted 45-minute, individual face-to-face interviews using a semi-structured interview guide to explore current practices, perceived issues, and broad individual expectations about elderly care. In this stage, the objective of the investigators conducting the interviews was to discuss the problems faced by each professional. The solutions per se would be more fully developed collectively in the focus groups that followed.

### Focus groups

Four focus groups were held in 90-minute sessions led by two researchers (IV, MDS). The multidisciplinary groups were held in parallel, and each group met four times. In the first session, the analysis of ideas on current practices and perceived issues, collected during the individual interviews, was presented to the group for discussion and in order to refine the findings. In the following sessions, participants were asked develop their expectations and propose solutions that would be acceptable to the group as a whole. The investigators performed an iterative analysis of the content from the focus groups, presenting the results at each successive session to refine the key features of the intervention. When the analyses revealed discrepancies, they were presented at the next focus group so that the issues could be resolved.

### Observation and documentation

Two researchers (IV, MDS) spent several days at various settings (hospitals, community-based health and social services) to observe and record representative or revealing practices. Documents (minutes, memos, activity reports) from each setting were also analyzed. These additional sources of information confirmed and complemented data gathered through interviews and focus groups.

### Data ordering, analysis, and definition of the key features of an intervention in elderly care

All individual interviews and focus groups were recorded and transcribed verbatim. Transcripts were produced, read, and coded by two of the researchers (IV, MDS), and validated by a third one (LL) to ensure that the resulting coding was not due to spurious associations. Transcripts were analyzed using standard methods of qualitative thematic analysis [36-39]. The process of iterative data analysis produced concepts and categories from which propositions emerged [36-39]. While the first iterations of the analysis were performed sequentially, the final analysis brought out key findings on issues and practices. These results were validated by the participants. Data gathered through observation and documentation were used to corroborate, validate, and complement the data obtained through interviews and focus groups. Indeed, the defining characteristics of the intervention were identified on the basis of current practices, perceived issues, and participants' expectations regarding elderly care [18,19].

## Results

Through this process, it was possible to define the features of an intervention in elderly care that met the professionals' and managers' expectations. Indeed, the stepwise approach comprised three steps, each of which led to specific results. In the first step of the pre-intervention study, we gathered data on practices, perceived issues, and broad expectations of healthcare professionals and managers. Participants shared the same perceptions regarding current practices and issues in elderly care. This step revealed the processes that lead to adverse outcomes and that needed to be improved through the intervention.

The second step involved multidisciplinary focus groups, which were held to define the characteristics of a customized intervention. Overall, the investigators' role in iterative data collection and focus group facilitation helped participants define the key objectives of the intervention. These key features were identified at the clinical, structural, and process levels.

**Figure 1:**
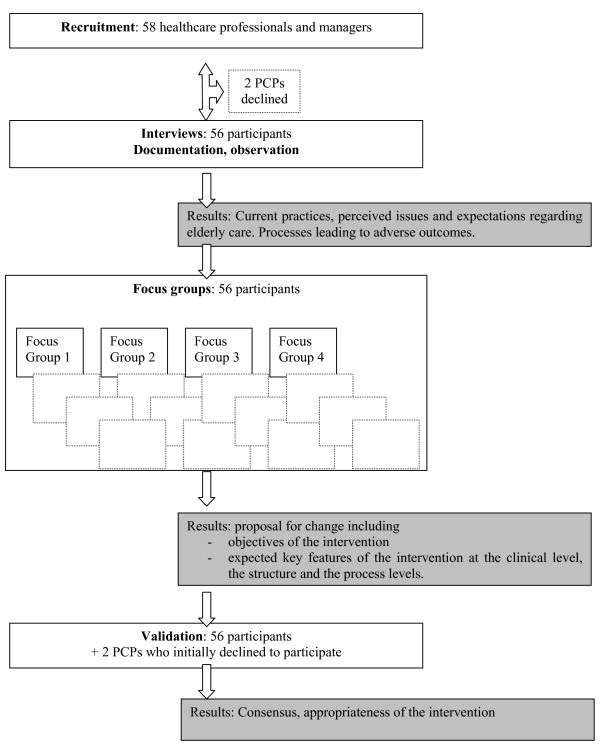
**Overall process from the pre-implementation study to the definition of key features of the intervention**.

The third step involved validating the data. A virtual consensus was reached on the current practices, issues, and key intervention features needed to respond to the identified issues. Indeed, in the validation step, of 56 participants, 53 'strongly agreed' or 'agreed' and three 'neither agreed nor disagreed' with the results. Subsequent interviews with the two PCPs who initially declined to participate confirmed that they agreed with the study findings and the key features of the intervention. The overall stepwise approach and the results of each step are described in Figure 1. Details of the final results are presented in the following sections and are summarized in Table 2 and Table 3.

**Table 2:** Managers' and healthcare professionals' current practices and perceived issues

Managers' and Healthcare Professionals' Current Practices and Perceived Issues
Challenges created by the complex and multidimensional chronic conditions of older persons

Primary Care Physician (PCP) as the key clinician

• Essential role of the PCP
• Older persons' loyalty to their PCPs
Inadequate needs assessment process within primary care

• Medical-centered. Lack of multidimensional needs assessment.
• Dichotomy between medical needs assessment/other assessments
Inadequate coordination of primary care services

• No one is responsible for coordinating services. PCPs' lack of time. Poor knowledge of services.
• Lack of communication between professionals.
• Fee-for-service remuneration
Inadequate coordination of primary and secondary care

• Poor planning of services at discharge
• Little continuity of care. No information on hospitalization provided to PCP
• Unavailability of direct hospitalization or geriatric expertise
Perceived Consequences:

• Unmet needs
• Inappropriate use of services. Unwanted institutionalization
• High family burden

**Table 3:** Key features of the proposed intervention in elderly care

Key features of the Proposed Intervention in Elderly Care	
Objectives:	• Improve care for older persons with complex and multidimensional chronic conditions
	• Prevent unwanted institutionalization and unnecessary use of services

Clinical, collaborative and organizational means:	Strengthen primary care
	• Maintain the PCP as the main medical practitioner
	• Integrate health professionals into a multidisciplinary team
	• Introduce a case manager collaborating with PCPs
	• No translocation to secondary care

	Coordinate primary and secondary care
	• Better communication
	• Access to geriatric expertise for PCP (a community-based geriatrician)
	• Direct access to hospitalization

	No change in funding mechanisms

### Current practices and perceived issues in elderly care

#### Main challenges

While caring for older persons in good health or with a single chronic problem seemed relatively straightforward to the participants, all participants mentioned the difficulties they encountered caring for 'their' very frail older persons with complex and multidimensional chronic conditions:

PCP D: 'Managing care for older people is complicated and time-consuming when they have a lot of problems. It's emotionally draining, it exposes your shortcomings.'

PCPs were identified as the key clinicians for frail elderly persons. PCPs felt responsible for their patients, and other participants confirmed this essential role, highlighting the loyalty felt by patients towards their PCPs:

PCP R: ' [As] the patient's family physician, I'm in a key position.

Home-care worker H: The woman felt close to this physician who didn't examine her. I told her to change, but she felt close to him. She trusted him.'

#### Inadequate needs assessment process within primary care

The participants agreed that the needs assessment process was not centered on common geriatric syndromes, but rather on acute medical problems. PCPs recognized that they were concentrating on the patients' complaints and the assessment of acute medical needs:

PCP C: 'We check to see if the problem is medical, but helping them and all that – we don't know how. There are geriatrics assessment sheets and forms, but we don't use them.'

Moreover, the assessment process did not employ a multidisciplinary approach. When other professionals (nurses, social workers, *et al*.) were involved, they performed their own needs assessments, which were not usually communicated to PCPs, creating incongruence between medical, functional, and social needs assessments:

Community-based nurse N: 'I'm quite aware when someone has difficulty breathing, when there's a change in their condition. I don't contact their physician directly, but I'll speak to the patient's wife about it.'

#### Inadequate coordination of primary care services

In practice, no one was responsible for coordinating services. PCPs often tried to play this role, but they did not have enough time and sufficient knowledge of existing services. Coordination problems were identified by all the participants, such as poor knowledge of each others' roles and poor communication and collaboration, particularly between social and health services:

Community-based social worker H: 'A woman with dementia was living with her daughter who could no longer handle all the responsibility. I would hope that [the PCP] would remember that home care services are available.'

Moreover, fee-for-service remuneration of PCPs and some other healthcare professionals was seen as one of the barriers to coordination, since the time they spent coordinating tasks was not compensated:

Community-based health service manager one: 'We need to know each other better. I'm glad I'm finally getting to see people in this meeting who I have only known by name.'

Community-based social service manager three: 'While the [PCP] is coordinating, he isn't with the patient, so he won't be paid (...). We can't get him to attend our meetings.'

#### Inadequate coordination of primary and secondary care

All participants found that inadequate coordination between primary and secondary care led to poor continuity of care. Hospital-based professionals acknowledged their poor knowledge of community-based services and the pressure to transfer patients quickly, which led to poor service planning at discharge and a lack of communication with community-based services:

Emergency physician B: 'We [hospital physicians] feel pressure over the length of hospital stays, and it results in not having the time to organize hospital discharges.'

Geriatrician H: 'The problem is that everyone works quite independently. When a patient returns home, sometimes it's just organized on the fly. We don't always know who was involved before the hospitalization.'

PCPs felt that access to hospital-based specialists, including geriatricians, was too complicated when they needed a consultation. Moreover, because PCPs were not routinely notified about patient discharges and decisions made during the hospitalization, it was difficult for them to make appropriate decisions after discharge:

PCP D: 'From time to time, we don't know what to do. (...) We don't know what occurred during the hospitalization... The hospital has no idea how we work. They've changed medications at the hospital, and we don't know why.'

#### Perceived consequences for patients and families

All participants felt that because of the problems identified, the overall needs of older persons were not being recognized or met in a timely manner, leading to 'crisis' situations. Consequently, while PCPs knew that an emergency room visit is an adverse experience for older patients (eg, long waits, use of restraints), they were still using it inappropriately (eg, falls, overextended families) because it was the only way for them to gain access to a geriatric assessment:

PCP A: 'After you've made four or five calls to the hospital and had no success or your request has been refused, you give up. We send them to the emergency room; at least we can be sure that they'll get a hospital bed.'

Moreover, transitions between settings were performed with insufficient exchange of information between clinicians. When the patients were discharged, their PCPs were not fully debriefed by the hospital, raising the risk of inappropriate care that would lead to a new crisis situation and a return to the emergency room. Hospital physicians were not clearly informed about the medical condition leading to the hospitalization, and they lacked information needed to make appropriate decisions. Poor coordination of care was therefore generating a vicious circle of emergency room visits and hospitalizations.

Finally, families were left too often with a significant burden. They tried to compensate for the lack of communication and coordination, but felt overwhelmed. When patients did not have family members to perform these coordination tasks, healthcare professionals had to consider institutionalization, even if the elderly patient wanted to be cared for at home:

Hospital social worker M: 'Most of the time, it's the service that gives the information to the family on how to complete the hospital discharge and apply for home services.'

Hospital nurse J: 'Before discharge, you need to determine if the family is ready to manage patient care. If the family is unavailable, if they work or live abroad, it won't work. So we look for an institutional placement.'

#### Defining characteristics of the intervention

The participants defined a proposal for change that included the objectives of the intervention and the key features needed to attain these objectives. More specifically, two main intervention objectives were deemed essential by all participants: improving quality of care for very frail older persons and preventing unnecessary hospital and emergency room use and unwanted institutionalizations:

PCP D: 'This is why our approach needs to change, so that we can provide better care and organize the care needed to keep patients in their homes.'

In order to meet these objectives, participants requested, first, that the intervention rely on multidisciplinary primary care and that the PCP remain the main medical practitioner. Participants felt that primary care should be strengthened by introducing an ongoing formal case-management process. This would include a multidimensional geriatric needs assessment, the development and implementation of care plans, coordination of services, and follow-up. This process would be supported by a multidisciplinary team of health professionals, with case managers collaborating closely with PCPs:

PCP S: 'If the case manager could take care of social problems and home care, that could help avoid hospitalizations, particularly if they can provide a rapid response (...).'

Second, participants requested the integration of primary and specialized care. Coordination between primary and specialized care needed to be improved through better service planning and better communication of relevant information at hospital discharge. Case managers would participate in the transition from hospital-based to community-based services. Moreover, PCPs expected to be informed of the care provided and decisions made during hospital stays. They wanted improved access to scientific evidence through the introduction of evidence-based protocols. In addition, they expected collaborative practices with geriatricians through the introduction of community-based geriatricians working as consultants, but they wanted to remain responsible for medical decision-making. PCPs would also be allowed to recommend direct hospital admissions rather than send their patients to emergency services:

PCP B: 'Easier access in order to hospitalize directly, without going through emergency. It's a question of trust with family physicians.'

Finally, the participants did not want any changes made to existing funding mechanisms for hospitals and community-based services:

Funding authority administrator two: 'The professionals are different, but so is the funding. And we aren't ready to combine budgets.'

## Discussion

The originality of this study lies in having systematically gathered data on current practices, issues, and expectations of healthcare professionals and managers in order to determine the main features of an intervention, which is generally recognized as a condition for successful implementation [19]. The results of the study suggest that it is feasible to determine the defining characteristics of an intervention that meets the expectations of healthcare professionals and managers. The detailed characteristics of the intervention, as well as a description of its successful implementation, have been presented in a previous report (Vedel I, De Stampa M, Bergman H, Ankri J, Cassou B, Mauriat M, Blanchard F, Bagaragaza E, Lapointe L: A novel model of integrated care for the elderly: COPA – Coordination of Professional Care for the Elderly, submitted). In the intervention group, 106 patients were recruited. They were 86.0 years old on average (S.D. 6.7) and represented a group of very frail elderly persons experiencing a mix of functional impairments, cognitive impairment, isolation, and medical conditions. Preliminary results from the quasi-experimental study suggest that elderly care was more appropriate during the intervention (as shown by a reduction in unnecessary health care service utilization), and that PCPs and nurses actively participated in the intervention and were satisfied with its design and implementation.

This pre-intervention study investigated the context of elderly care, which is recognized in implementation research as particularly important and challenging [40,41]. The study identified current practices and issues in elderly care and the processes that lead to the adverse outcomes often described in the literature, such as inappropriate use of hospital services [40,41], poor quality of medical care provided to community-dwelling older patients [42], family burdens [43], and inappropriate decisions made by PCPs after discharge [44].

Beyond providing a portrait of current practices and processes that lead to adverse outcomes, the results of the study helped researchers design a new intervention to improve elderly care. Researchers did not use formal methods to select the key features of the intervention. Rather, they focused on the solutions to elderly care issues suggested by the participants [45]. Indeed, their role was to synthesize the solutions proposed by the focus group and present them to the following focus group. When discrepancies emerged, they were presented to the focus groups as questions with the goal of refining the key features of the intervention. This iterative approach to data collection and focus group facilitation allowed participants to enter into progressively more detailed discussions of the issues in elderly care and gradually work out the key features of the intervention. It was an approach that both addressed the issues and took current practices into account.

The results of the study suggested that the intervention should focus on three levels: the individual level, such as the implementation of evidence-based protocols; the team level, such as the implementation of collaborative practices; and the organizational level, such as the integration of services. These results highlight the importance of intervening at different levels, including at the organizational level and not solely at the individual level. While intervening at different levels – changing the behaviour of individual clinicians but also the structure and the process of care – is generally recognized as appropriate in most of the contexts and particularly in chronic care [1,5,20,46,47], this approach is rarely methodically explored through pre-intervention studies such as the one conducted here [22].

Moreover, the results of the study suggest that two points deemed essential to the participants have not received sufficient attention in interventions in elderly care. First, interventions should not only focus on older persons with specific pathologies such as dementia [48] or congestive heart failure [49], but also address all older people with complex chronic conditions. Second, this study has highlighted the fact that secondary services are seriously failing to respond to the expectations of primary care professionals. Even if, as it is often pointed out in the literature, reorganizing primary care is essential [42] in order to respond to the poor quality of primary care [4,50], primary care professionals – and particularly by PCPs – clearly also expect an improvement in coordination between primary and secondary care.

The overall strategy we used had led to a high participation rate and the development of a virtual consensus among participants. The participants deemed it essential to adopt a broader approach when developing an intervention in the context of elderly care, both in terms of the population (frail elderly with complex medical conditions) as well as in terms of the reorganization of care (not only reorganizing primary care but also the primary/secondary care interface). At the end of the process, nearly all participants agreed with the key features of the intervention, including the two PCPs who initially declined to participate in the qualitative study. This final agreement provided a strong argument for the appropriateness of the intervention in the sense that it responded to the characteristics of the context and to the professionals' expectations. Several factors can explain these results. First, the individual interview phase allowed participants to understand that the goal was to develop an intervention that would address concrete problems. This may explain why they continued to participate through all the stages of the study (interviews, focus groups, and validation), despite the significant amount of time that this required. Indeed, involving professionals in the intervention development process may reduce their resistance, enhance their motivation, and encourage the kind of culture of change that is essential for improving quality and safety in healthcare [51].

Second, solutions were not discussed in the individual interview stage as a way of ensuring that the key features of the intervention would be developed in the multidisciplinary focus groups. Indeed, the use of focus group methods to develop an intervention allows participants to share their points of view. This type of local and social interaction offers the best chances for a successful dissemination of change as well as a reduction in perceived barriers in general [52] and in healthcare in particular [19].

The data in this study reflected local issues, so the potential for generalizing these findings is limited. However, the qualitative method provided insights into current practices, issues, expectations, and directions for developing appropriate interventions. Another limitation of this study was the absence of data collection from the elderly. Unfortunately, the great majority of the patients targeted by this intervention (disabled elderly persons who were 85 years old, on average) suffer from major cognitive disorders, and this ruled out interviews. In addition, we could not replace such an interview with an interview with their family because of poor concordance rates, particularly in cases where the elderly patient suffered from dementia [53,54]. We decided to focus this extensive study on the professionals' views in order to develop an intervention based on current practices and the expectations of the professionals. The design therefore featured all the hospitals and community-based health and social services in a geographic zone (an arrondissement of Paris), which allowed insights to be gathered from health care professionals and managers working in various settings. Finally, a limitation of the tailoring method presented in this article is the amount of time spent before the implementation of the intervention, whereas the participants may have preferred a quick intervention that would have addressed their problems. The investigators played a key role, carefully customizing the intervention to the issues. They often had to remind participants that the issues had to be thoroughly analyzed before any attempts could be made at developing solutions, and that the goal was to work together to find solutions that would be acceptable to the group. The length of this pre-intervention study (2.5 years) can only be understood in terms of the complexity of the intervention and the need to have so many types of professionals and professional settings involved in the process. In situations where the intervention is less complex, however, a pre-intervention study would not need to be as long as the one described here. When proven interventions are available, even if the barriers to their implementation need to be identified [45], it is not be necessary to develop all the key features of the intervention, and a shorter pre-intervention study will probably suffice.

Multiple coding by two researchers (IV, MDS) provided added rigor. A third researcher (LL) validated the analysis and played the role of critical reviewer to establish evidence that the findings were not the result of spurious associations. In order to enhance the internal validity of the data, four sources of evidence were used: interviews, focus groups, observation, and documentation. The participants iteratively reviewed the findings, which left them open to scrutiny and challenge and enhanced their validity. All participants – including the two PCPs who initially refused to participate to the study – validated the final results.

## Conclusion

This article presents an innovative strategy in the intervention design process. We performed a preliminary qualitative study of the practices and expectations of healthcare professionals and managers and thus defined the characteristics of an intervention that would meet the professionals' and managers' expectations. The results of the study suggest that this strategy was feasible and could provide new information on the expected characteristics of the intervention in the context of elderly care. This study provides an example of a method that can be used to perform a pre-intervention study to determine the defining features of an intervention customized to the context of care. The method should be tested in other healthcare settings with other populations. Further research will be needed in order to develop a more thorough understanding of the impact of these strategies on intervention implementations.

## Competing interests

The authors declare that they have no competing interests.

## Authors' contributions

IV, HB, and LL designed the study. IV and MDS developed and conducted the structured interviews. IV, MDS, and LL analyzed all the interviews. All authors read and approved the final manuscript.
